# The Study's Chemical Interaction of the Sodium Silicate Solution with Extender Pigments to Investigate High Heat Resistance Silicate Coating

**DOI:** 10.1155/2021/5510193

**Published:** 2021-04-05

**Authors:** Cuong Manh Le, Thu-Huong Le

**Affiliations:** ^1^Faculty of Building Material, National University of Civil Engineering, Hanoi 100000, Vietnam; ^2^Faculty of Chemistry and Environment, Thuyloi University, Hanoi 100000, Vietnam

## Abstract

Silicate coating is water-based paint with many advantages and wide applications in many different industries. However, there are still some problems with silicate coating: how to increase its resistance to heat at high temperatures and prolong the life of the coating. Silicate paints have high durability and longevity dependent mainly on the chemical interaction of the silicate binder with extender pigments. Therefore, our groups have studied the geopolymerization process of the sodium silicate solution with extender pigments to investigate high heat resistance silicate coating. The effect of curing time on the chemical interaction between sodium silicate solution and extender pigments (ZnO, TiO_2_, Fe_2_O_3_, CaCO_3_, and Na_2_SiF_6_) was investigated by Fourier transform infrared spectroscopy (FT-IR), thermal gravimetric analysis (TGA), and X-ray diffraction (XRD). The shift of antisymmetric stretching vibration of the Si-O-Si bond (1060 cm^−1^) to low frequency and increase of the intensity of the Si-O-Si stretching as curing time increases from 1 to 20 days are due to the increased chemical interaction between extender pigments (ZnO, TiO_2_, Fe_2_O_3_, CaCO_3_, and Na_2_SiF_6_) and sodium silicate solution. Moreover, TG results of ZnO-silicate, TiO_2_-silicate, CaCO_3_-silicate, Na_2_SiF_6_-silicate, and Fe_2_O_3_-silicate coating at 1 and 20 days of curing show high residual geopolymer about 69–90% at 800°C. Thus, we proposed that the geopolymerization process between sodium silicate solution and extender pigments (ZnO, TiO_2_, Fe_2_O_3_, CaCO_3_, and Na_2_SiF_6_) increases when the curing time from 1 to 20 days leads to forming geopolymer silicate with high thermal stability. In addition, the optimal mixing ratio between sodium silicate solution and extender pigments (ZnO, TiO_2_, Fe_2_O_3_, CaCO_3_, and Na_2_SiF_6_) is as follows: 25% binder (sodium silicate solution), 8% ZnO; 5% TiO_2_, 5% Fe_2_O_3_, 1% Na_2_SiF_6_, 21% CaCO_3_, 34% H_2_O, and 1% additives to make high heat resistance silicate coating with temperature resistance at 1000°C.

## 1. Introduction

Vietnam is a country that has a tropical climate with high humidity and high temperature. Moreover, there are many impurities in the air such as dust, microorganisms, and toxic chemicals. With the above climate, many types of building materials are damaged, mossy, and rusty. Coating technology is used as a film to protect buildings from chemical attack and corrosion of the environment. There are two commonly used paints, organic and inorganic coating [1]. The organic coating such as epoxy resin shows good mechanical performance, chemical resistance, and anticorrosive effect [2]. However, it has been shown that organic coating has many disadvantages such as high aging and especially high price [3]. Besides, organic-inorganic hybrid materials are materials in which organic (epoxy resin) and inorganic (rice husk silica [4, 5], nano-glass fiber, and silanized coal fly ash [6]) components are mixed to enhance the mechanical and fracture toughness of epoxy resin. The inorganic coating is the main component of cross-linked inorganic binders such as silicon resin [7], water- and solvent-based silicates [8], silanes [9], and a mixture of the organic binder with silicate [10]. In comparison with organic coating, inorganic coating (silicate coating) shows more advantage because of its corrosion and heat resistance [11]. However, there are still some problems with silicate coating such as long-term durability, low resistant water, and high price, which have limited the wide commercial applications of organic coating in the construction and building industry [12].

The need to address issues to develop and deploy in the practice of new silicate paint materials required the development of new approaches to developing silicate coating. Herein, recent achievements in the modified properties and working mechanism of silicate coating by adding organic and inorganic compounds make the silicate coating even more promising for the application. Yuan et al. reported a study on the properties and working mechanism of the waterborne polyurethane-modified silicate-based coating. The bending, compressive strengths, and flexibility of the coating modified by adding polyurethane were greatly improved compared with the unmodified coating. The temperature resistance of the waterborne polyurethane-modified silicate-based coating is low at 250°C [13]. Parashar et al. reported about alkali silicate-based zinc-rich coating with excellent abrasion resistance, prevention of undercutting of coatings by corrosion, unaffected by temperatures up to 528°C, and unaffected by the organic solvents such as ketones and gasoline [14]. Bahreini's group also synthesized silica supported ZnO nanoparticle as the new pigment for thermal control coating [15]. Besides, Irfan Khan et al. have used TiO_2_ pigment to improve the photocatalytic activity of silicate paint [16]. However, the silicate paint which is the suspension of extender pigments (Zn, ZnO nanoparticle, and TiO_2_) in liquid potassium glass has some disadvantages, for example, low flexibility and high cost. The liquid sodium glass (sodium silicate solution) is of interest for use as a binder for silicate coating, which ensures more high-performance properties and low cost [12]. The use of liquid sodium glass does not provide the water-resistance of silicate coatings [12]. Extender pigments are of interest for use as filler for silicate paints that ensure more high-performance properties. Moreover, silicate coating has high heat resistance, durability, and longevity dependent mainly on the curing process of the silicate binder with extender pigments. Therefore, understanding the interaction of liquid glass with extender pigments and colorants will help us to create paint products that are heat resistant and of high durability and longevity. Silicate coating with sodium silicate film is a new direction due to its high heat resistance, long life, and low price and completely suitable for the climatic conditions of Vietnam. To investigate the effects of the interaction of liquid glass with extender pigments on the properties of silicate coating, our groups have studied the curing process of sodium silicate solution with each of the extender pigments such as ZnO, TiO_2_, CaCO_3_, Na_2_SiF_6_, and Fe_2_O_3_. The chemical interaction between sodium silicate solution and extender pigments according to curing time was investigated by FT-IR, TGA, and XRD. In addition, the novel material based on extender pigments (ZnO, TiO_2_, CaCO_3_, Na_2_SiF_6_, and Fe_2_O_3_) was prepared to investigate the high heat resistance silicate coating. The thermal stabilization of silicate coating was investigated by TGA and SEM.

## 2. Materials and Methods

### 2.1. Chemicals

Sodium silicate solution (mNa_2_O.nSiO_2_.xH_2_O, *d* = 1.35 g/cm^3^), zinc oxide (ZnO, ≥99%), titanoxide (TiO_2_, ≥99%), calcium carbonate (CaCO_3_, ≥99%), ferric oxide (Fe_2_O_3_, ≥99,5%), and sodium silicon fluoride (Na_2_SiF_6_, ≥99.5%) were purchased from Merk.

### 2.2. Experimental

#### 2.2.1. Preparation of Extender Pigments (ZnO, TiO_2_, Fe_2_O_3_, CaCO_3_, and Na_2_SiF_6_) Silicate Coating

To study the curing process of sodium silicate solution with extender pigments at 1 day and 20 days, each of the extender pigments (ZnO, TiO_2_, Fe_2_O_3_, CaCO_3_, and Na_2_SiF_6_) was added to the 5.5–6.5 g sodium silicate solution and stirred at room temperature until getting powder (note that the amount of the extender pigments was added to sodium silicate solution so that the drying time of the mixture to get dry powder is less than 20 minutes). The coating powders were crushed by the ball milling method for 1 hour; after that the coating powder was cured from 1 to 20 days in the air under room temperature. Therefore, the formula of the mixture of liquid sodium glass with each of the extender pigments shows in Table 1.

The effect of curing time on the chemical interaction between sodium silicate solution and extender pigments (ZnO, TiO_2_, Fe_2_O_3_, CaCO_3_, and Na_2_SiF_6_) was investigated by Fourier transform infrared spectroscopy (FT-IR), thermal gravimetric analysis (TGA), and X-ray diffraction (XRD). Fourier transform infrared spectroscopy (FT-IR) measurements were conducted on a Nicolet 380 spectrometer (Waltham, MA, USA) operated in the mid-IR range of 4000–400 cm^−1^, with spectra obtained at a spectral resolution of 8 cm^−1^ in transmittance mode. Thermogravimetric analysis (TGA) and derivative thermogravimetry (DTG) were performed using a METTLER TOLEDO SDTA851e. The coating powder at 1 and 20 days of curing was placed in aluminum oxide (AlO_x_) TGA pan. The sample was heated at rate of 10°C min^−1^ from 20 to 900°C under a nitrogen gas flow of 50 mL.min^−1^. X-ray diffraction (XRD) spectra of absorption materials were obtained using D8 Advance (Bruker-Germany) and D5005 (Siemens- Germany).

#### 2.2.2. Preparation of the Silicate Coating

**Scheme 1:**
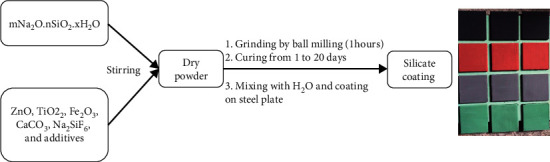
Preparation of the silicate coating.

The silicate coating was prepared (Scheme 1) by mixing sodium silicate solution with extender pigments (ZnO, TiO_2_, Fe_2_O_3_, CaCO_3_, Na_2_SiF_6_, and additives) and stirred at room temperature for 20 minutes until getting dry powder by following the formula in Table 2. The dry silicate coating powders were crushed by ball milling for 1 hour; after that the coating powder was cured from 1 to 20 days. The silicate coating sample at 20 days of curing was mixed with water and coated on the steel plate.

The silicate coating was prepared (Scheme 1) by following the formula in Table 2. The role of ZnO, TiO_2_, and Fe_2_O_3_ in silicate paints is to enhance the mechanical properties of the paint such as acid strength, heat resistance, and leaching resistance of the silicate coating due to chemical interaction with liquid glass to form the geopolymer. Na_2_SiF_6_ is added to the paint to increase the leaching resistance of the paint film due to the chemical interaction between Na_2_SiF_6_ and liquid sodium silicate by the following reaction:

(1) $$\begin{array}{c} {\text{Na}}_{2}{\text{SiF}}_{6}+2\left({\text{Na}}_{2}\text{O}{\text{.SiO}}_{2}+{\text{H}}_{2}\text{O}\right)=6\text{NaF}+{3\text{SiO}}_{2}{\text{.H}}_{2}\text{O} \end{array}$$

CaCO_3_ is the primary filler of most silicate coatings, because a part of CaCO_3_ interacts with the liquid sodium glass to form calcium silicate that is both mechanically stable and water resistant.

The thermal stabilization of the silicate coating was investigated by TGA and scanning electron microscopy (SEM). The SEM was performed with S-4800 (SEM, Hitachi) at an accelerating voltage of 30 kV. The heat resistance of silicate coating was investigated by calcination of the paint film at 1000°C for 10 h.

## 3. Results and Discussion

The chemical interaction between sodium silicate solution and extender pigments (ZnO, TiO_2_, Fe_2_O_3_, CaCO_3_, and Na_2_SiF_6_) results are as follows.

**Figure 1:**
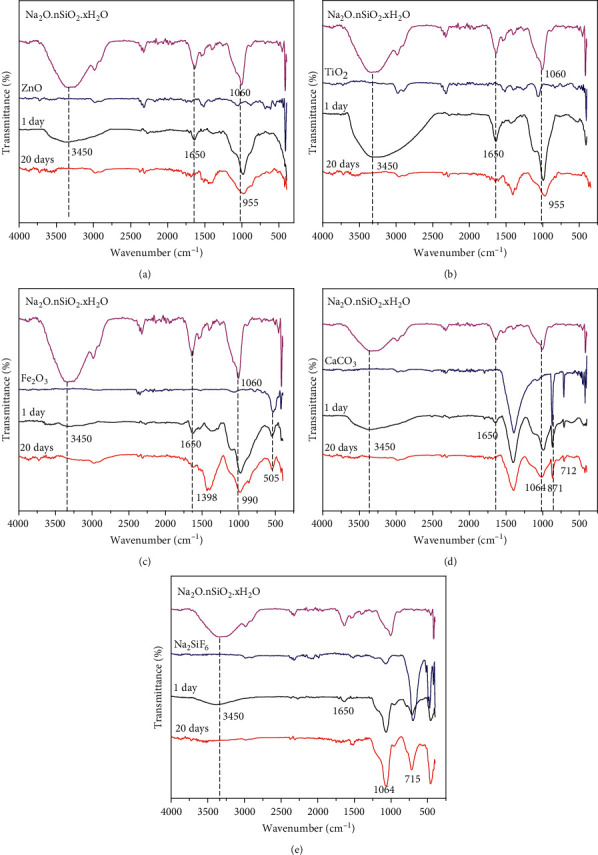
FT-IR spectra of ZnO-silicate (a), TiO_2_-silicate (b), Fe_2_O_3_-silicate (c), CaCO_3_-silicate (d), and Na_2_SiF_6_-silicate coating (e) at 1 day and 20 days of curing compared with extender pigments (ZnO, TiO_2_, Fe_2_O_3_, CaCO_3_, and Na_2_SiF_6_) and Na_2_O.nSiO_2_.xH_2_O.

The FT-IR spectra of extender pigments-silicate coating (Figures 1(a)–1(e)) show the variation in the chemical structure according to the curing time from 1 day to 20 days. The ZnO-silicate, TiO_2_-silicate, CaCO_3_-silicate, Na_2_SiF_6_-silicate, and Fe_2_O_3_-silicate coating at 1 day of curing's sample show the peaks at 3450 and 1650 cm^−1^, indicating the presence of the asymmetric stretching and bending of hydroxyl (-OH) groups from silanol (Si-O-H) groups or residual water on the surface of the sample [16]. When the curing time increased to 20 days, the intensity peak at 3450 cm^−1^ decreased due to the dehydration and dehydroxylation to form silicate geopolymer.

In Figures 1(a)–1(b), the FT-IR spectra of Na_2_O.nSiO_2_.xH_2_O show the peak at 1060 cm^−1^, indicating the presence of the Si-O-Si stretching vibration [17]. However, the FT-IR spectrum of ZnO-silicate (a) and TiO2-silicate coating (b) samples with curing time of 1 and 20 days shows the peaks at 955 and 960 cm^−1^, respectively. The vibration shifted to the low frequency at 955 cm^−1^ as sodium silicate solution mixed with ZnO, indicating the formation of ZnO–SiO_2_ bond [18–20]. Lee et al. and Zhang et al. have determined the chemical structure of SiO_2_/TiO_2_ composite by FT-IR spectrum. The FT-IR results of SiO_2_/TiO_2_ composite show vibration at 925 cm^−1^, which is assigned to the formation of Ti-O-Si bonds [21, 22]. Therefore, we suggest that the shift of the antisymmetric stretching vibration (1060 cm^−1^) of the Si-O-Si bond to the low frequency (955 and 960 cm^−1^) and the decrease in the intensity of Si–OH bending vibration of sample of 20 days of curing compared with 1-day sample were because of the complete geopolymerization process between sodium silicate solution and ZnO and TiO_2_ to form the chemical bonds Si-O-Zn and Ti-O-Si [16, 17, 20].

The chemical bonds between the Fe_2_O_3_ and sodium silicate solution at curing time of 1 and 20 days (Figure 1(c)) were further investigated using FT-IR spectra. In Figure 1(c), the absorption band near 500 and 1398 cm^−1^ of Fe_2_O_3_-silicate coating samples at 1 day and 20 days of curing are assigned to Fe-O stretching mode, indicating the presence of the Fe_2_O_3_ in the coating samples [23]. The peak at 1060 cm^−1^ corresponding to Si-O-Si stretching vibration of Na_2_O.nSiO_2_.xH_2_O shifts to the low frequency at 990 cm^−1^ with increasing curing time from 1 to 20 days, which is assigned to the formation of the Fe-O-Si bond [24]. Therefore, we suggest that the shift of the antisymmetric stretching vibration of the Si-O-Si bond (1060 cm^−1^) to low frequency at 990 cm^−1^ and the decrease in the intensity of Si–OH bending vibration of sample of 20 days of curing compared with 1-day sample are because of the complete geopolymerization process between sodium silicate solution and Fe_2_O_3_ to form of new chemical bonds such as Fe-O-Si.

The FT-IR spectra of CaCO_3_-silicate coating (Figure 1(d)) at 1 day and 20 days of curing compared with FT-IR spectra of CaCO_3_ and Na_2_O.nSiO_2_.xH_2_O show the variation in the chemical structure according to curing time from 1 to 20 days. The FT-IR results of samples of 1 and 20 days reveal that the absorption peak at 1060 cm^−1^ confirms the formation of silicate (SiO_2_) due to the chemical interaction between CaCO_3_ and liquid sodium silicate. The absorption peaks at 712, 871, and 1460 cm^−1^ are attributed to the C-O bond out-of-plane bending, in-plane bending, and stretching vibrations of CO_3_^2-^, respectively [25, 26]. In addition, the intensity peak at 1060 cm^−1^ which is related to the constitutional SiO_2_ increases as the curing time increases from 1 to 20 days [16, 17]. Therefore, when the curing time increases from 1 to 20 days, the geopolymerization process between CaCO_3_ and liquid sodium silicate is more complete due to the chemical interaction between CaCO_3_ and liquid sodium silicate according to the following reaction:

(2) $$\begin{array}{c} \left({\text{Na}}_{2}\text{O}{\text{.nSiO}}_{2}+{\text{H}}_{2}\text{O}\right)+{\text{CaCO}}_{3}={\text{Na}}_{2}{\text{CO}}_{3}+\left(n-1\right){\text{SiO}}_{2}+{\text{H}}_{2}\text{O}+{\text{CaSiO}}_{3} \end{array}$$

In Figure 1(e), FT-IR spectra of Na_2_SiF_6_-silicate coating at 1 day and 20 days of curing compared with Na_2_SiF_6_ and Na_2_O.nSiO_2_.xH_2_O show the variation in the chemical structure according to curing time from 1 to 20 days. The FT-IR results of samples at 1 day and 20 days of curing reveal that the absorption peak at 1060 cm^−1^ of SiO_2_ is due to the chemical interaction between Na_2_SiF_6_ and liquid sodium silicate [16, 17]. In addition, the intensity peak at 1060 cm^−1^ which is related to the constitutional SiO_2_ increases as the curing time increases from 1 to 20 days. Therefore, when the curing time increases from 1 to 20 days, the geopolymerization process between Na_2_SiF_6_ and liquid sodium silicate is more complete due to the chemical interaction between Na_2_SiF_6_ and liquid sodium silicate according to the following reaction:

(3) $$\begin{array}{c} {\text{Na}}_{2}{\text{SiF}}_{6}+2\left({\text{Na}}_{2}\text{O}{\text{.SiO}}_{2}+{\text{H}}_{2}\text{O}\right)=6\text{NaF}+{3\text{SiO}}_{2}{\text{.H}}_{2}\text{O} \end{array}$$

The FT-IR spectrum of extender pigments-silicate coating (Figure 1) shows the peak at 1060 cm^−1^ corresponding to Si-O-Si stretching vibration of Na_2_O.nSiO_2_.xH_2_O shift to low frequency about 900–960 cm^−1^ when sodium silicate solution is mixed with extender pigments (ZnO, TiO_2_, and Fe_2_O_3_). Besides, the intensity peak at 1060 cm^−1^ of silicate (SiO_2_) due to the chemical interaction between extender pigments (CaCO_3_ and Na_2_SiF_6_) and sodium silicate solution increases as the curing time increases from 1 to 20 days. Therefore, the geopolymerization process between extender pigments (ZnO, TiO_2_, Fe_2_O_3_, CaCO_3_, and Na_2_SiF_6_) and sodium silicate solution to form new bonds suggested provoking the significant changes observed in the shift to the low frequency of antisymmetric stretching vibration of the Si-O-Si bond (1060 cm^−1^) and increase of the intensity of the Si-O-Si stretching as curing time increases from 1 to 20 days.

### 3.1. X-Ray Diffraction Pattern

**Figure 2:**
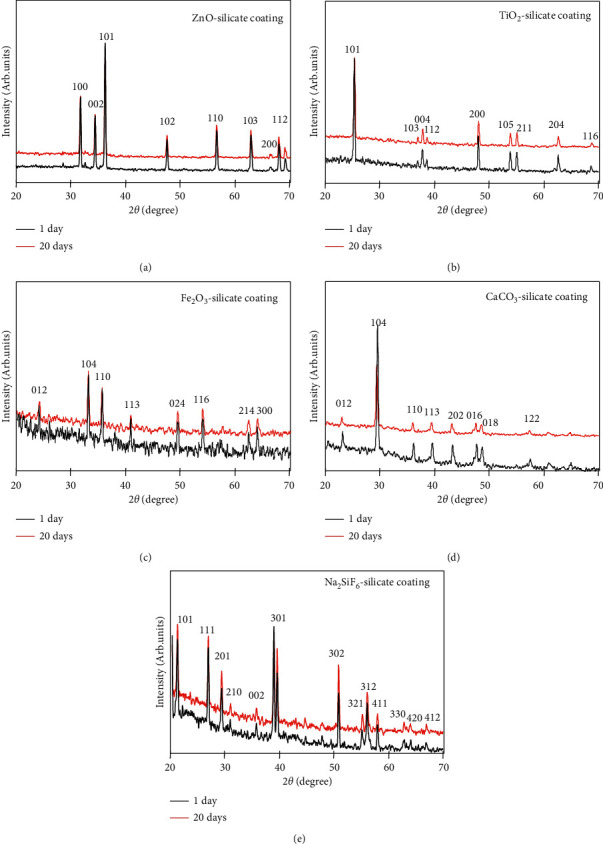
Powder X-ray diffraction pattern of the of ZnO-silicate (a), TiO_2_-silicate (b), Fe_2_O_3_-silicate (c), CaCO_3_-silicate (d), and Na_2_SiF_6_-silicate coating (e) at 1 day and 20 days of curing.

The crystallinity of extender pigments-silicate coating (Figures 2(a)–2(d)) at 1 day and 20 days was determined by X-ray diffraction (XRD). In Figures 2(a)–2(d), the XRD results of extender pigments-silicate coating at 1 and 20 days of curing show the peaks correspond to the ZnO with wurtzite phase (JCPDS 79-2205) [27], TiO_2_ with anatase phase (JCPDS-21-1272) [28], *α*-Fe_2_O_3_ phase (JCPDS card No: 79–0007) [29], CaCO_3_ (JCPDS card number 47–1743) [30], and Na_2_SiF_6_ with hexagonal phase (JCPDS no. 33–1280) [31]. Also, the intensity peaks of 20 days of curing's sample are lower than the intensity peaks of 1 day of curing's sample due to the chemical interaction between extender pigments and liquid sodium silicate. Therefore, we propose that the chemical reaction of sodium silicate solution and extender pigments (ZnO, TiO_2_, Fe_2_O_3_, CaCO_3_, and Na_2_SiF_6_) increases when the curing time increases from 1 to 20 days resulting in more completing of the geopolymerization process.

### 3.2. Thermal Analysis

**Figure 3:**
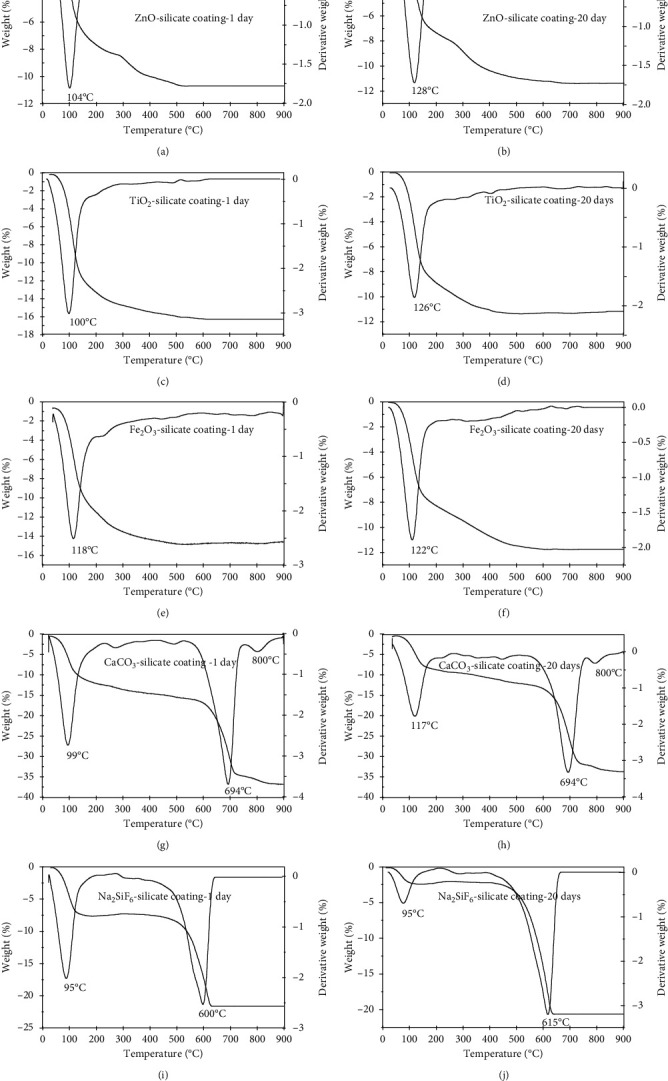
TGA and DTG curves of the stable mixing of ZnO-silicate coating at 1 day (a); ZnO-silicate coating at 20 days (b); TiO_2_-silicate coating at 1 day (c); TiO_2_-silicate coating at 20 days of curing (d); Fe_2_O_3_-silicate coating at 1 day (e); Fe_2_O_3_-silicate coating at 20 days (f); CaCO_3_-silicate coating at 1 day (g); CaCO_3_-silicate coating at 20 days (h); Na_2_SiF_6_-silicate coating at 1 day (i); Na_2_SiF_6_-silicate coating at 20 days of curing (j).

Thermal stability of extender pigments-silicate coating was investigated by thermal gravimetric analysis. TG and DTG curves at 10°C/min heating rate for 1 and 20 days of curing were given in Figure 3. As shown in Figure 3, the total residual geopolymer of 1 and 20 days of curing's samples is 89 and 90% for ZnO-silicate coating, 84 and 89% for TiO_2_-silicate coating, 64 and 69% for CaCO_3_-silicate coating, 71 and 79,5% for Na_2_SiF_6_-silicate coating, and 85 and 88% for Fe_2_O_3_-silicate coating at 800°C, respectively. Besides, the DTG plots of ZnO-silicate, TiO_2_-silicate, Fe_2_O_3_-silicate, CaCO_3_-silicate, and Na_2_SiF_6_-silicate coating show the increase in dehydration temperature of surface water (physically and chemically absorbed water) when curing time increases from 1 to 20 days. Drying or dehydration of surface water occurred below 100°C, whereas fixed water was released about 95–118°C for 1 day of curing and 95–128°C for 20 days of curing's samples. This increase in dehydration temperature resulted from the increase of interaction of water molecules and geopolymer.

The DTG plots of ZnO-silicate coating at 1 and 20 days of curing (Figures 3(a) and 3(b)) show that the peak at 315°C of ZnO-silicate coating at 1 and 20 days of curing represents the dehydration of bonded water (Si-O-H) process. The ZnO-silicate coating at 1 and 20 days of curing shows that the small peak at 500°C that accounts for 2–5% of the mass loss (1 day of curing) and the one at 650°C that accounts for 1–2% of the mass loss (20 days of curing) are considered due to the melting of silica catalyzed by metal impurities (Na^+^) and formation of bulk silicate [32].

The DTG plots of CaCO_3_-silicate coating at 1 and 20 days of curing (Figures 3(g) and 3(h)) show that the peak at 694°C of CaCO_3_-silicate coating at 1 and 20 days of curing's samples represents the thermal decomposition of CaCO_3_ process according to the following reaction:

(4) $$\begin{array}{c} {\text{CaCO}}_{3}⟶\text{CaO}+{\text{CO}}_{2} \end{array}$$

The CaCO_3_-silicate coating at 1 and 20 days of curing shows the small peak at 800°C that accounts for 2–5% of the mass loss due to the melting of silica catalyzed by metal impurities (Na^+^ and Ca^2+^) and formation of bulk silicate [32].

The DTG plots of Na_2_SiF_6_-silicate coating at 1 and 20 days of curing (Figures 3(i) and 3(j)) show that the peak at 600°C of Na_2_SiF_6_-silicate coating at 1 day and the one at 615°C of Na_2_SiF_6_-silicate coating at 20 days of curing's samples represent the decomposition of Na_2_SiF_6_ according to the following reaction:

(5) $$\begin{array}{c} {\text{Na}}_{2}{\text{SiF}}_{6}⟶\text{NaF}+{\text{SiF}}_{4} \end{array}$$

We proposed that the increased decomposition temperature of Na_2_SiF_6_ as curing time increases from 1 to 20 days is due to the increased chemical reaction between Na_2_SiF_6_ and sodium silicate solution binder.

TG results of ZnO-silicate, TiO_2_-silicate, CaCO_3_-silicate, Na_2_SiF_6_-silicate, and Fe_2_O_3_-silicate coating at 20 days of curing show high residual geopolymer about 69–90% at 800°C. The DTG plots show the increase in dehydration and thermal decomposition temperature resulted as the curing time increases from 1 to 20 days. As the discussion in FT-IR results, the shift of antisymmetric stretching vibration of the Si-O-Si bond (1060 cm^−1^) to low frequency and increase of the intensity of the Si-O-Si stretching as curing time increases from 1 to 20 days are due to the chemical interaction between extender pigments (ZnO, TiO_2_, Fe_2_O_3_, CaCO_3_, and Na_2_SiF_6_) and sodium silicate solution to form new bonds. Therefore, we proposed that the geopolymerization process between sodium silicate solution and extender pigments (ZnO, TiO_2_, Fe_2_O_3_, CaCO_3_, and Na_2_SiF_6_) increases when the curing time increases from 1 to 20 days leading to forming geopolymer silicate with high thermal stability. And the newly introduced material based on extender pigments was expected to give a high heat resistance silicate coating, which is very useful for applications in devices working at high temperatures.

### 3.3. Silicate Coating

**Figure 4:**
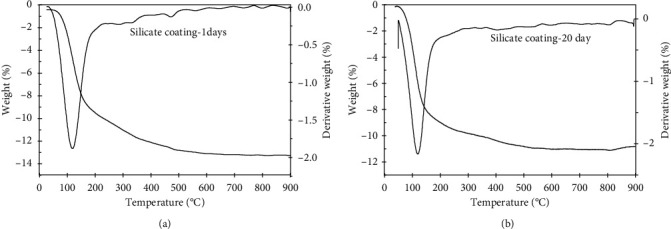
TGA and DTG curves of the stable mixing of silicate coating sample at 1 day (a); TGA and DTG curves of the stable mixing of silicate coating sample at 20 days (b).

The thermal stability of the silicate coating was investigated by thermal gravimetric analysis. TGA and DTG curves at 10°C/min heating rate for 1 and 20 days of curing are given in Figures 4(a) and 4(b). As shown in Figures 4(a) and 4(b), the total residual geopolymer of 1 and 20 days of curing's samples is 87% and 88.5% at 900°C, respectively. Besides, the DTG plots of 1 and 20 days of curing show that the drying or dehydration of surface water occurred at 103°C for 1 day of curing's sample and 108°C for 20 days of curing's sample. This increase in dehydration temperature resulted from a high heating rate (10°C/min) and the interaction of water molecules and geopolymer. When the curing time increases from 1 to 20 days, the geopolymerization process between sodium silicate solution and extender pigments (ZnO, TiO_2_, CaCO_3_, Na_2_SiF_6_, and Fe_2_O_3_) is more completed. Thus, we choose the silicate coating sample at 20 days of curing for testing thermal stability (at 1000°C).

**Figure 5:**
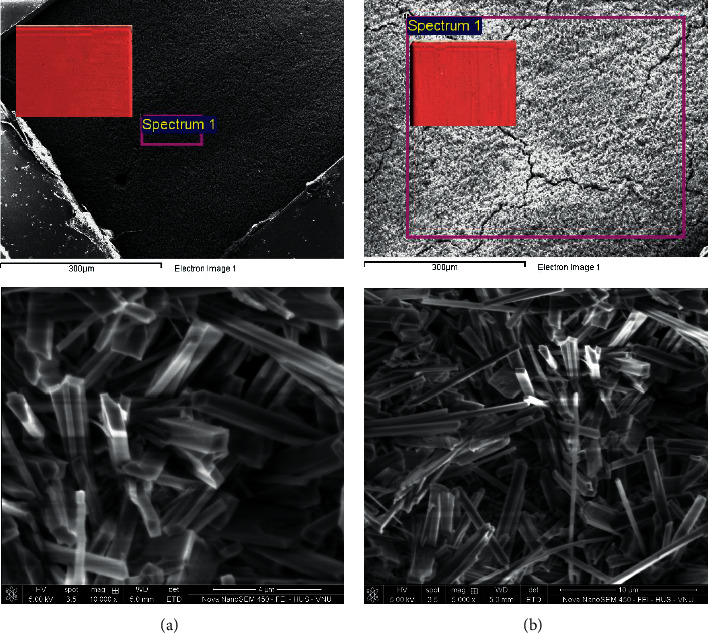
SEM image results of the silicate coating film (curing at 20 days) on the steel plate before (a) and after heating at 1000°C (b).

SEM image results (Figure 5) of the silicate coating film before (a) and after heating at 1000°C (curing at 20 days) showed that the surface morphology after calcination of the silicate coating on the steel plate at 1000°C did not have much difference compared to the silicate coating on the steel plate before calcination. The needle shape morphology was found in the microstructural analysis of geopolymers in this study. In particular, the surface and color of the film that can be seen with the naked eye do not change after calcination at 1000°C for 10 h. Hence, the silicate paint is of high heat resistance and completely suitable for the climatic conditions of Vietnam.

## 4. Conclusion

In this report, we have investigated the effect of curing time on the chemical interaction between sodium silicate solution and each of the extender pigments such as ZnO, TiO_2_, CaCO_3_, Na_2_SiF_6_, and Fe_2_O_3_ by FT-IR, XRD, and TGA. The geopolymerization process between extender pigments (ZnO, TiO_2_, Fe_2_O_3_, CaCO_3_, and Na_2_SiF_6_) and sodium silicate solution to form new bonds suggested provoking the significant changes observed in the shift of antisymmetric stretching vibration of the Si-O-Si bond (1060 cm^−1^) to low frequency and increase of the intensity of the Si-O-Si stretching as curing time increases from 1 to 20 days. TGA results of mixture between extender pigments (ZnO, TiO_2_, Fe_2_O_3_, CaCO_3_, and Na_2_SiF_6_) and sodium silicate solution at 1 and 20 days of curing show high residual geopolymer about 69–90% at 800°C. Also, the optimal mixing ratio between sodium silicate solution and extender pigments such as ZnO, TiO_2_, Fe_2_O_3_, CaCO_3_, and Na_2_SiF_6_ is as follows: 25% binder (sodium silicate solution), 8% ZnO, 5% TiO_2_, 5% Fe_2_O_3_, 1% Na_2_SiF_6_, 21% CaCO_3_, 34% H_2_O, and 1% additives to make high heat resistance silicate coating with temperature resistance at 1000°C. Thus, the newly introduced material based on extender pigments was expected to give a high heat resistance silicate coating, which is very useful for applications in devices working at high temperatures.

## Figures and Tables

**Table 1:** The components of the mixture of liquid sodium glass with each of the extender pigments (ZnO, TiO_2_, Fe_2_O_3_, CaCO_3_, and Na_2_SiF_6_).

	The components of the coating	Weight percent (%)
The coating powder 1	mNa_2_O.nSiO_2_.xH_2_O	55
	ZnO	35
	H_2_O	10

The coating powder 2	mNa_2_O.nSiO_2_.xH_2_O	55
	TiO_2_	35
	H_2_O	10

The coating powder 3	mNa_2_O.nSiO_2_.xH_2_O	55
	CaCO_3_	35
	H_2_O	10

The coating powder 4	mNa_2_O.nSiO_2_.xH_2_O	65
	Na_2_SiF_6_	15
	H_2_O	10

The coating powder 5	mNa_2_O.nSiO_2_.xH_2_O	55
	Fe_2_O_3_	35
	H_2_O	10

**Table 2:** The components of the silicate coating.

The components of silicate coating	Weight percent (%)
mNa_2_O.nSiO_2_.xH_2_O	25
ZnO	8
TiO_2_	5
Fe_2_O_3_	5
Na_2_SiF_6_	1
CaCO_3_	21
H_2_O	34
Additives	1

## Data Availability

All the data and supporting materials are included within the article.
